# Pneumatic wound compression after hip fracture surgery

**DOI:** 10.3109/17453670903317114

**Published:** 2009-10-01

**Authors:** Torsten Johansson

**Affiliations:** Division of Orthopaedics, Department of Clinical and Experimental Medicine, Faculty of Health Sciences, Linköping UniversitySwedentorsten.johansson@lio.se

*Sir*—I have read with interest the article by Dr. Apelqvist et al. In their well-performed study they did not find pneumatic wound compression to reduce the need for blood transfusion after hip fracture surgery. In the discussion the authors mention several explanations for not receiving positive results found in two previous studies concerning the same wound compression device used after elective total hip arthroplasty (Hörnberg et al. 2002, Johansson 2005a). Of course, a mix of hip fracture patients, operated on by different methods, differs from patients with hip osteoarthritis operated on with a total hip arthroplasty. However, the authors do not comment on the fact that all patients in their study received tranexamic acid. Tranexamic acid is probably the most powerful, simplest and most cost-effective treatment in minimizing blood loss after a total hip arthroplasty (Johansson et al. 2005b). This strong effect may even overshadow the possible positive effects of wound compression for hip fracture patients.
