# Supplementary material for: Pneumatic wound compression after hip fracture surgery
Source: Acta Orthop. 2009 Oct 1;80(5):628. doi: 10.3109/17453670903317114 (PMC2823324; doi:10.3109/17453670903317114)
Supplement: Related texts

# Sub article

**Published:** 2009-10-01

**Authors:** Anna Apelqvist, Markus Waldén, Gert-Uno Larsson, Isam Atroshi

**Affiliations:** Department of Orthopedics Hässleholm-Kristianstad, Hässleholm HospitalSwedenanna.apelqvist@skane.se

*Sir*—We thank Dr Johansson for his comment. The use of tranexamic acid was already in our clinical routine in hip fracture surgery and we made no exception for this study. Our trial was performed to evaluate the efficacy of the compression bandage in further reducing the need for postoperative blood transfusion. Of course we can not know whether the results would have been any different had tranexamic acid not been given prior to surgery. However, this seems unlikely since the small differences in mean blood units and in proportion of patients transfused, though statistically insignificant, tended to be in favor of the non compression group for all 3 surgical procedures. The mean number of blood units was in the compression group 1.3 and in the non-compression group 1.1. Blood transfusion was given to 84 (62%) in the compression group and to 85 (55%) in the non-compression group.
